# *Leviviricetes*: expanding and restructuring the taxonomy of bacteria-infecting single-stranded RNA viruses

**DOI:** 10.1099/mgen.0.000686

**Published:** 2021-11-08

**Authors:** Julie Callanan, Stephen R. Stockdale, Evelien M. Adriaenssens, Jens H. Kuhn, Janis Rumnieks, Mark J. Pallen, Andrey N. Shkoporov, Lorraine A. Draper, R. Paul Ross, Colin Hill

**Affiliations:** ^1^​ APC Microbiome Ireland, University College Cork, Cork, Co. Cork, T12 YT20, Ireland; ^2^​ Quadram Institute Bioscience, Norwich Research Park, Norwich, NR4 7UQ, UK; ^3^​ Integrated Research Facility at Fort Detrick, National Institute of Allergy and Infectious Diseases, National Institutes of Health, Frederick, Maryland 21702, USA; ^4^​ Latvian Biomedical Research and Study Centre, Rātsupītes 1, Riga, LV-1067, Latvia; ^5^​ University of East Anglia, Norwich, UK; ^6^​ School of Veterinary Medicine, University of Surrey, Guildford, UK

**Keywords:** *Leviviricetes*, levivirus, metatranscriptomics, phage, virus classification, virus taxonomy

## Abstract

The vast majority of described prokaryotic viruses have double-stranded or single-stranded DNA or double-stranded RNA genomes. Until 2020, a mere four prokaryotic single-stranded, positive-sense RNA viruses have been classified in two genera (*Riboviria; Lenarviricota; Allassoviricetes; Leviviridae*). Several recent metagenomic and metatranscriptomic studies revealed a vastly greater diversity of these viruses in prokaryotic soil communities than ever anticipated. Phylogenetic analysis of these newly discovered viruses prompted the reorganization of class *Allassoviricetes*, now renamed *Leviviricetes*, to include two orders, *Norzivirales* and *Timlovirales*, and a total of six families, 428 genera and 882 species. Here we outline the new taxonomy of *Leviviricetes*, approved and ratified in 2021 by the International Committee on Taxonomy of Viruses, and describe open-access hidden Markov models to accommodate the anticipated identification and future classification of hundreds, if not thousands, of additional class members into this new taxonomic framework.

## Data Summary

The taxonomic proposal (TaxoProp) on the reorganization and renaming of lenarviricot class *Allassoviricetes* (TaxoProp 2020.095B.R.Leviviricetes), written by the authors of this article [1] and subsequently ratified by the International Committee on Taxonomy of Viruses (ICTV) in March 2021 [2], can be found at ICTV (ictvonline.org).

To support the continued expansion of the newly organized class *Leviviricetes* within phylum *Lenarviricota*, hidden Markov models (HMMs) generated from the expanded number of available bacteria-infecting positive-sense single-stranded RNA (+ssRNA) virus proteins are available at Figshare: https://doi.org/10.6084/m9.figshare.12745394.v1.

The authors confirm all supporting data, code and protocols have been provided within the article or through supplementary data files.

**Impact Statement**

This work describes a taxonomic framework for the classification of bacteria-infecting positive-sense, single-stranded RNA (+ssRNA) viruses and is the largest proposal submitted to the Bacterial Viruses Subcommittee of the International Committee on Taxonomy of Viruses (ICTV) to date. Both metagenomic-sourced sequences and known isolates of +ssRNA viruses were incorporated into a taxonomic scheme using novel algorithms and automated systems. The existing hierarchical organization of bacteria-infecting +ssRNA viruses was overhauled to capture their vast diversity uncovered through metagenomic approaches and these viruses are now unified in the class *Leviviricetes*. Furthermore, this work presents a case study adopting a Latinised binomial species nomenclature for all 882 species as a standardized nomenclature for all viruses. Looking forward, the methods and resources developed in this study support future phylogenetic analyses and taxonomic classifications of newly isolated viruses and metagenomic viral sequences.

## Introduction

The genome of the positive-sense single-stranded RNA (+ssRNA) phage MS2, which infects *Escherichia coli* bacteria, was the first of any biological entity ever to be sequenced [3]. In the 1971 First Report of the International Committee on Nomenclature of Viruses (ICNV; today the International Committee on Taxonomy of Viruses [ICTV]), phage MS2 was classified as a member of the ‘ribophage group’ [4], which in the Second Report (1976) was considered a genus in the then-new family Leviviridae [unitalicized at the time] [5]. Through the discovery of additional *E. coli* +ssRNA phages, the family was slightly expanded. Most recently (prior to 2021), family *Leviviridae* included two genera for a total of four viruses. Those four viruses remained the only classified viruses in *Leviviridae*-including class *Allassoviricetes –* one of four classes in phylum *Lenarviricota* (Table 1), although some 50 other viruses were considered possible members of the family. This low diversity starkly contrasts that of the thousands of prokaryotic viruses, predominantly with double-stranded (ds) DNA, but also with single-stranded (ss) DNA and dsRNA, genomes which are classified across five of the currently six established virus realms (*Adnaviri*a, *Duplodnaviria*, *Monodnaviria*, *Riboviria* and *Varidnaviria*) [6, 7].

**Table 1.** Pre-2021 taxonomy of +ssRNA bacterial viruses [6, 8, 24]

Realm	Kingdom	Phylum	Class	Order	Family	Genus	Species	Virus
*Riboviria*	*Orthornavirae* (one of two kingdoms in the realm)	*Lenarviricota* (one of five phyla in the kingdom)	*Allassoviricetes* (one of four classes in the phylum)	*Levivirales*	*Leviviridae*	*Allolevivirus*	*Escherichia virus F1*	Enterobacteria phage FI 4184 b
							*Escherichia virus Qβ*	Escherichia phage Qbeta
						*Levivirus*	*Escherichia virus BZ13*	Escherichia phage BZ13
							*Escherichia virus MS2*	Escherichia phage MS2

The genomes of all viruses previously classified in *Leviviridae* contain three core genes encoding a maturation protein (MP), a coat protein (CP) and a catalytic subunit of an RNA-directed RNA polymerase (RdRP), respectively; leviviruses were distinguished from alloleviviruses by additional encoding a lysis protein as opposed to a unique ‘read-through’ protein [8]. Whereas the classified four viruses have been isolated in culture, these hallmark genes and the polarity of the virus genome can be used for relatively straightforward identification of related viruses in sequence datasets. Recently such metagenomic and metatranscriptomic datasets have become available. Their focused analysis revealed that previous virome studies were frequently methodically biassed against identification of phage MS2-like viruses, such studies focusing on the preservation of DNA, purification of virus-like particles with dsDNA genomes, and failing to incorporate the RNA fraction in their analyses. Once these biases were removed, a plethora of these viruses became apparent, indicating that bacteria-infecting +ssRNA viruses are much more abundant and diverse than previously thought [9–14]. This discovery triggered a rigorous and fundamental overhaul of the taxonomy of class *Allassaviricetes* and resulted in a more robust taxonomic framework that will aid future progress in our understanding of bacteria-infecting +ssRNA virus diversity, evolution and ecological significance.

### Profile HMMs to detect and classify bacteria-infecting +ssRNA viruses

**Fig. 1.**
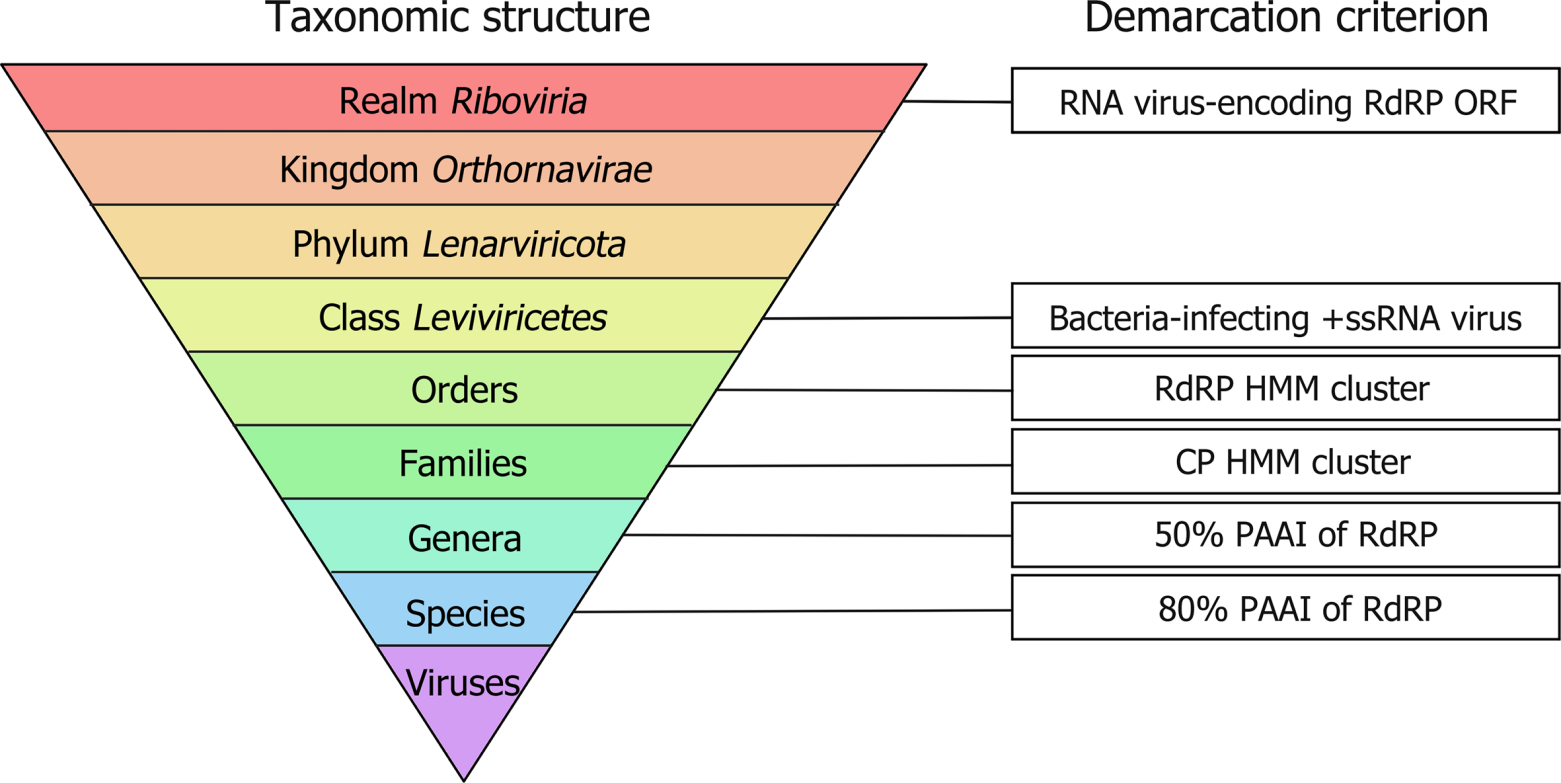
Taxon demarcation criteria for leviviricetes classification. Taxonomic ranks for positive-sense single-stranded RNA (+ssRNA) viruses are shown alongside the demarcation criterion for each of the taxon ranks. PAAI, pairwise amino-acid sequence identity; CP, coat protein; RdRP, RNA-directed RNA polymerase; HMM, hidden Markov model; ORF, open reading frame.

**Fig. 2.**
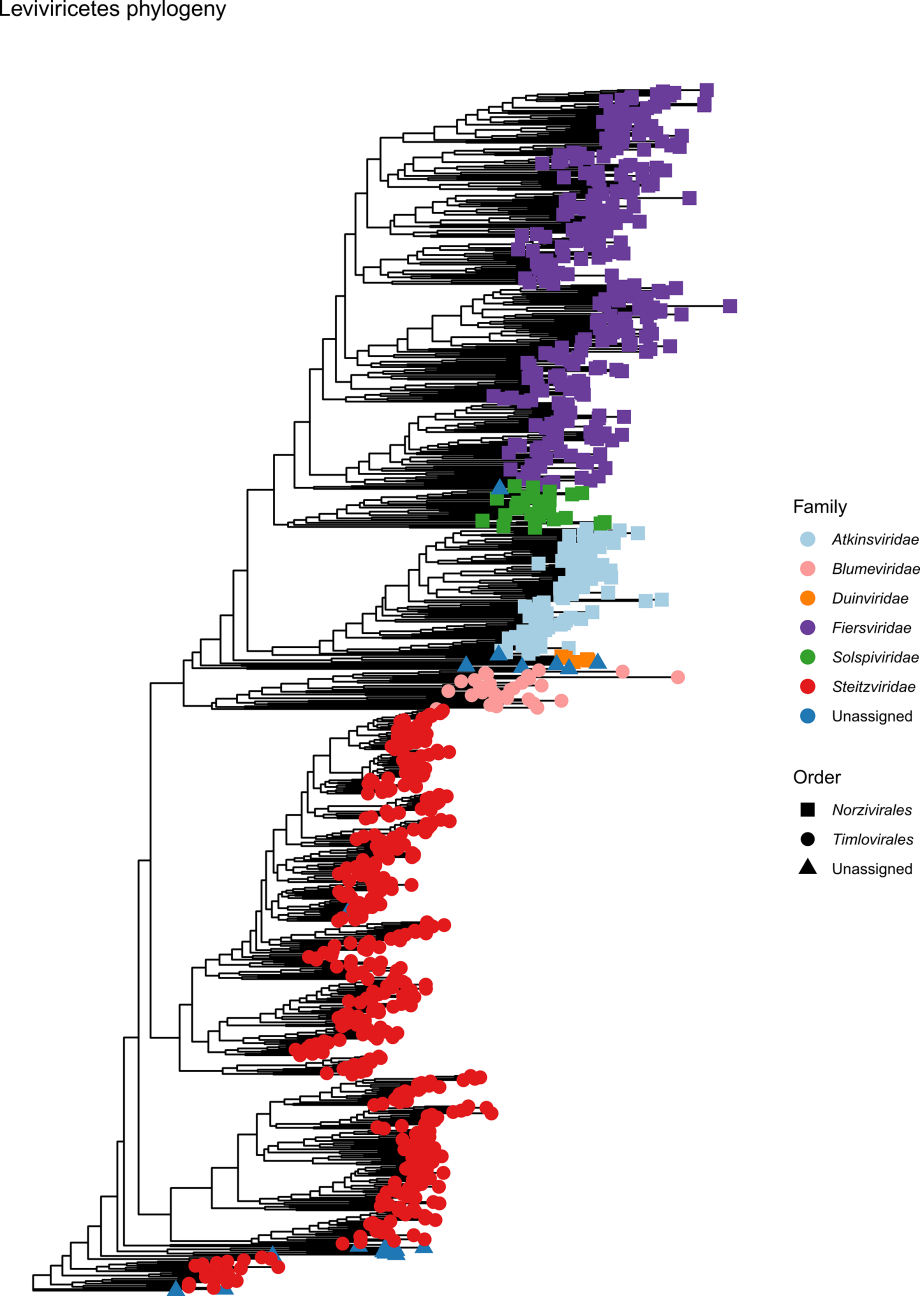
HMM taxonomic predictions of *Leviviricetes*. While expanding and restructuring positive-sense single-stranded RNA (+ssRNA) viruses, nine genera could not be assigned with confidence to a family or an order, as the RNA-directed RNA polymerase (RdRP) and coat protein (CP) genome-encoded combinations did not adhere to established combinations. Additionally, there were ten instances (out of 882 species representative sequences) for which the HMM predicted taxonomy of +ssRNA viruses did not align with their phylogeny-based assignment. The colour and shape aesthetics of the phylogenetic tree illustrates these taxonomic outliers.

To create a novel taxonomic framework for bacteria-infecting +ssRNA viruses, 1868 genomic sequences tentatively identified as such in [10, 12–14] were obtained from the US National Center for Biotechnology Information (NCBI). Grouping of proteins encoded by these viruses was achieved using orthoMCL, which implements Markov clustering [15]. Nine CP clusters, three MP clusters and two RdRP clusters were generated and their alphabetical labelling reflects their original descriptions [12]. Profile hidden Markov models (HMMs) based on orthoMCL clusters were used to detect distant relationships among the three proteins (CP, MP, RdRP). The results of phylogenetic analysis of RdRPs and CPs overall agreed with protein clustering. Therefore, the evolutionary relationships in phylogenies of the RdRPs and CPs were used as the demarcation criteria for establishing orders and families, respectively (Fig. 1). A comparison of the phylogenetic and open-access HMM approaches (see Data Summary) for bacteria-infecting +ssRNA virus classification yielded only ten instances (out of 882 species-representing sequences) of disagreements (Fig. 2). Therefore, although not perfect in its classification predictions, the HMMs will confidently identify bacteria-infecting +ssRNA virus sequences and provide end users with additional information to continue the expansion of the taxonomic framework presented here.

## Taxonomy of class *leviviricetes*

A comparison of the pre-2021 and the 2021 taxonomic breakdowns of bacteria-infecting +ssRNA viruses highlights the significant expansion and restructuring of the *Leviviricetes* taxon by incorporating metagenome-assembled genomes (Tables 1–3). Taxa established at the order and family ranks are named after prominent +ssRNA virus biologists. The co-discoverers of +ssRNA viruses are acknowledged in the generation and assignment of the two order names, whereas family names were randomly assigned to +ssRNA virus scientists irrespective of the viruses classified at these taxonomic ranks. The description of orders and families are presented alphabetically and do not reflect the historical or future predicted contributions of specific scientists to the +ssRNA virus field. As the *Leviviricetes* taxon is adjusted over time, newly established ranks do not necessarily need to continue the presented naming system.

**Table 2.** 2021 taxonomy of +ssRNA bacterial viruses [1, 24, 25]

Realm	Kingdom	Phylum	Class	Order	Family	Genus	Species	Virus
*Riboviria*	*Orthornavirae* (one of two kingdoms in the realm)	*Lenarviricota* (one of five phyla in the kingdom)	*Leviviricetes* (one of four classes in the phylum)	*Norzivirales* (one of two orders in the class)	*Fiersviridae* (one of six families in the order)	*Qubevirus*	*Qubevirus faecium*	Enterobacteria phage FI 4184 b
							*Qubevirus durum*	Escherichia phage Qbeta
						*Emesvirus*	*Emesvirus japonicum*	Escherichia phage BZ13
							*Emesvirus zinderi*	Escherichia phage MS2

**Table 3.** Numerical summary of the 2021 taxonomy of +ssRNA bacterial viruses (*Riboviria*; *Orthornavirae; Lenarviricota*) [1, 24, 25]

Class	Orders	Families	Coat protein (CP) clusters	No. of genera included in family	No. of species included in family
*Leviviricetes*	*Norzivirales*	*Atkinsviridae*	C	56	91
		*Duinviridae*	AP205-like	6	6
		*Fiersviridae*	A, B and H	185	298
		*Solspiviridae*	G	24	31
	*Timlovirales*	*Blumeviridae*	E	31	35
		*Steitzviridae*	D and F	117	412
	Unassigned	Unassigned	n/a	9	9

### Class

The previously established class *Allassoviricetes* was renamed as *Leviviricetes* (a de facto elevation of former family *Leviviridae* to class rank) to retain the *Levi*- word stem, reflecting the colloquial use of the term ‘levivirus(es)”. This class now includes all +ssRNA viruses encoding the specific pattern of three +ssRNA virus core proteins: MP, CP and RdRP. In a recent analysis of +ssRNA virus genomes, 1868 sequences fit this genome architectural criterion. Additionally, the encoded MP and RdRP were required to meet a minimum length threshold of 350 and 500 amino acid residues, respectively, to ensure only near-complete (coding-complete) genomes were investigated. The 1868 sequences originated from sequences available through the National Center for Biotechnology Information (NCBI) and the studies of Callanan *et al*., Starr *et al*., Shi *et al*. and Krishnamurthy *et al*. [10, 12–14].

### Orders

We adopted clustering and separation of the RdRP into distinct phylogenetic clades as the order demarcation criterium because the RdRP is the most conserved protein across leviviricetes with the strongest phylogenetic signal [12]. Orders were named after prominent +ssRNA virus biologists.

The order *Norzivirales* (formerly named *Levivirales*) is based on the phylogeny and clustering of bacterial +ssRNA virus RdRP protein sequences. It is named after Norton Zinder (1928–2012), who isolated the first bacterial virus with an RNA genome and who continued to make crucial findings regarding these entities [16, 17]. A total of 426 bacteria-infecting +ssRNA viral species representatives are categorized as belonging to the *Norzivirales* order. Tying in with its original description, the profile HMM output additionally describes *Norzivirales* hits as cluster RdRP_A [12].

*Timlovirales*: This order is based on the phylogeny and clustering of bacterial +ssRNA virus RdRP protein sequences (cluster RdRP_B). It is named after Timothy Loeb (1935–2016) who, with Norton Zinder, isolated the first +ssRNA bacterial virus [16]. There are 447 leviviricetes classified in *Timlovirales*.

### Families

Familial taxonomic groups were based on the distinct phylogeny of bacterial +ssRNA virus CP sequences, as either a single cluster or collection of clusters generated from orthoMCL [12]. Out of 882+ssRNA virus species representatives, there were nine instances for which the phylogeny of the CP cluster did not match its predicted corresponding RdRP cluster; no order or familial taxonomic rank was designated for these +ssRNA viruses. Once additional related viruses to these outliers are identified, it will be possible to resolve their taxonomy, which may require the formation of additional families. The families *Atkinsviridae*, *Duinviridae*, *Fiersviridae* and *Solspiviridae* are the new families created within the *Norzivirales* order, whereas *Blumeviridae* and *Steitzviridae* are the new families in the *Timlovirales* order.

*Atkinsviridae* is named after John Atkins (1944–present) for his discovery of the lysin protein from Escherichia virus MS2 [18]. This family encompasses +ssRNA viruses predicted to encode a CP corresponding to CP cluster C (HMM profile CP_C). There are 91 viruses classified within *Atkinsviridae*.

*Blumeviridae* is named after Thomas Blumenthal (1943–present) for his findings on the replication of bacterial +ssRNA viruses, in particular the structure and function of the replicase [19]. This family encompasses +ssRNA viruses predicted to encode a CP corresponding to CP cluster E (HMM profile CP_E). Currently, 35 +ssRNA viruses are classified within *Blumeviridae*.

*Duinviridae* is named after Jan van Duin (1937–2017) for his discoveries related to novel bacterial +ssRNA viruses, and the RNA folding within bacterial +ssRNA virus genomes to control gene expression [20, 21]. This family encompasses +ssRNA viruses predicted to encode a CP corresponding to CP cluster AP205-like. Six leviviricetes are classified within *Duinviridae*.

*Fiersviridae* is named after Walter Fiers (1931–2019) who sequenced the first gene and genome of any organism, MS2, previously assigned to the species *Escherichia virus MS2* (3). This family encompasses +ssRNA viruses predicted to encode a CP corresponding to CP clusters A, B and H (HMM profiles CP_A, CP_B, and CP_H, respectively). There are 298 viruses currently assigned to *Fiersviridae*.

*Solspiviridae* is named after Sol Spiegelman (1914–1983), who discovered an RNA chain of only 218 nucleotides that could be reproduced by an RdRP [22]. This family encompasses +ssRNA viruses predicted to encode a CP corresponding to CP cluster G. There are 31 viruses classified within the *Solspiviridae* family (HMM profile CP_G).

*Steitzviridae* is named after Joan Argetsinger Steitz (1941–present) for her determination of an initiation sequence that is central to modern-day ribosome profiling [23]. This family encompasses +ssRNA viruses predicted to encode a CP corresponding to CP clusters D and F (HMM profiles CP_D and CP_F, respectively). A total of 412 bacteria-infecting +ssRNA viruses are classified within *Steitzviridae*.

### Genera

**Fig. 3.**
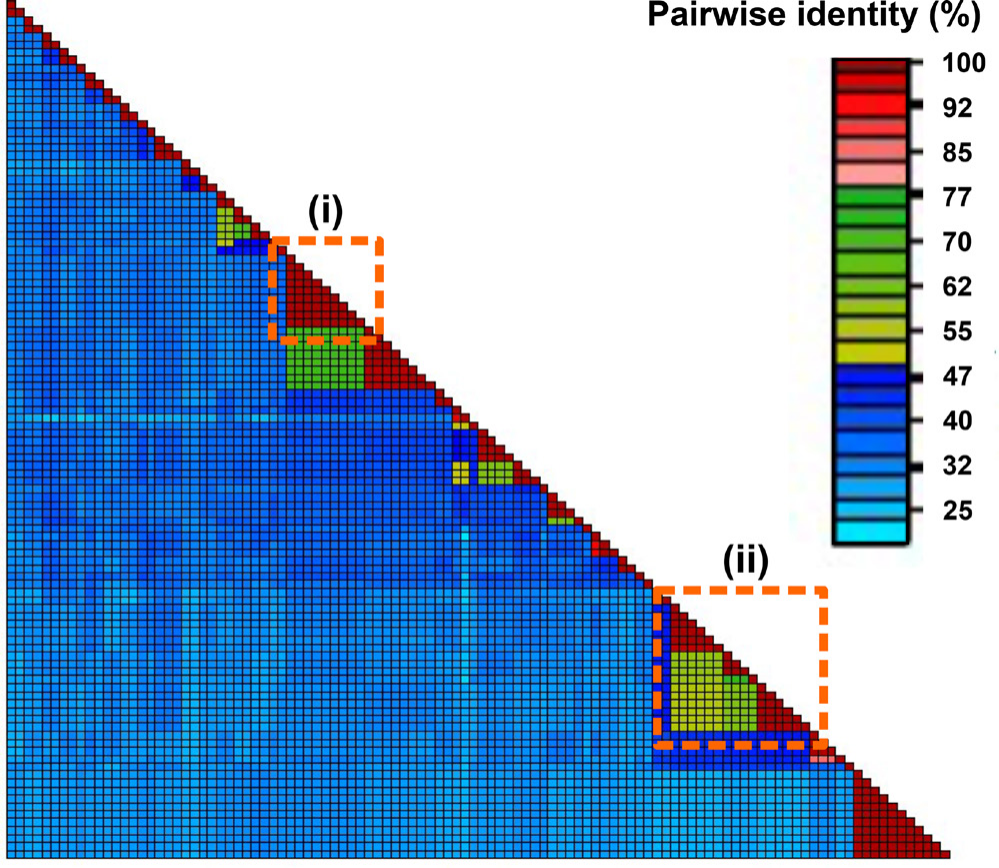
Examples of *Leviviricetes* genus and species demarcation cut-offs of 50 and 80 %, respectively, applied to pairwise RNA-directed RNA polymerase (RdRP) amino-acid sequence comparisons for members of norziviral *Atkinsviridae*. Inset (i) shows a distinct species clustering (red colouring), whereas inset (ii) shows three species represented by multiple sequences, and a species representing a single sequence, clustered into a genus (yellow-green colouring). Pairwise comparisons in shades of blue do not meet the set genus or species clustering criteria.

We chose 50 % pairwise amino-acid identity (PAAI) of the viral encoded RdRP as the criterion for establishing genera based on an analysis of the previous ICTV classification of known bacteria-infecting +ssRNA viruses (Fig. 3). Exemplar viruses representing each of the 428 genera were chosen by the following criteria, in decreasing priority: A bacterial +ssRNA virus representing the genus was chosen if (i) it was a previously described bacterial +ssRNA virus available in the ICTV archives, (ii) its sequence had been deposited in GenBank, (iii) or its contig was the longest of all remaining available sequences. The full list of genera included in each family can be found at ICTV (ictvonline.org) [24].

### Species

We chose 80 % PAAI of the RdRP as the species demarcation criterion (Fig. 3). This cut-off yielded 882 species, with each sequence assigned to a specific species included in a single genera. Species were named following a Latinized binomial species name format in compliance with the latest International Code of Virus Classification and Nomenclature (ICVCN) iteration [2]. The full list of species included in each genus can be found at ICTV (ictvonline.org) [24]. For example, phage MS2 is now assigned to the species *Emesvirus zinderi* and phage BZ13 is now assigned to *Emesvirus japonicum*, whereas phage Qbeta is assigned to *Qubevirus durum* and FI 4184 b is assigned to *Qubevirus faecium*. The new naming scheme no longer necessitates knowledge of host bacteria and is therefore well-suited to the incorporation of sequence-only or uncultured virus genomes.

## Discussion

The massive expansion in the discovery of novel bacteria-infecting +ssRNA virus genomes is now complemented with a timely update to their associated taxonomy. Fitting with phage MS2 being the first organism to have its genome completely sequenced, the presented ICTV-approved *Leviviricetes* taxonomic proposal detailed here is the first to systematically include metagenomic sequences to build a class-rank taxonomy incorporating automatic approaches. This effort supports incorporation of metagenomic sequences within ICTV’s framework in future taxonomic proposals and subsequent expansion of established virus taxonomic groups – thus advancing a holistic understanding of viral diversity. At present, the expansion and restructuring of *Leviviricetes* has been one of the largest-ever proposals submitted to and approved by the ICTV. However, as the incorporation of metagenome-assembled genomes into ICTV taxonomic proposals become more frequent, due to the immense unexplored diversity of the virosphere, this record will likely be short-lived.
